# Fibrin glue delivery system containing rhein ameliorates intervertebral disc degeneration by anti-inflammatory efficacy

**DOI:** 10.1186/s13018-023-03961-9

**Published:** 2023-07-06

**Authors:** Jianhang Bao, Wenshuo Gao, Wei Zhang, Dong Wang, Hao Pan

**Affiliations:** 1 Department of Orthopaedics, Hangzhou TCM Hospital Affiliated to Zhejiang Chinese Medical University (Hangzhou Hospital of Traditional Chinese Medicine), No. 453 Tiyuchang Road, Xihu District, Hangzhou, 310007 Zhejiang Province People’s Republic of China; 2 Department of Orthopaedics, Hangzhou Dingqiao Hospital, No. 1630 Huanding Road, Shangcheng District, Hangzhou, 310021 Zhejiang Province People’s Republic of China; 3 Institute of Orthopaedics and Traumatology, Hangzhou TCM Hospital Affiliated to Zhejiang Chinese Medical University, No. 453 Tiyuchang Road, Xihu District, Hangzhou, 310007 People’s Republic of China

**Keywords:** Drug delivery system, Rhein, Fibrin gel, Pyroptosis, Intervertebral disc degeneration

## Abstract

**Purpose:**

To construct an injectable, sustained-release fibrin gel containing rhein to solve the problem of low bioavailability of rhein, and observe its efficacy in the treatment of intervertebral disc degeneration.

**Methods:**

The fibrin gel containing rhein was first synthesized in advance. Subsequently, the materials were characterized by various experimental methods. Secondly, the degenerative cell model was constructed by stimulating nucleus pulposus cells with lipopolysaccharide (LPS), and the corresponding intervention treatment was carried out to observe the effect in vitro. Finally, the rat tail intervertebral disc was acupunctured by needles to establish the intervertebral disc degeneration model, and the effect of the material was observed through intradiscal injection.

**Results:**

The fibrin glue containing rhein (rhein@FG) showed good injectability, sustained release and biocompatibility. Rhein@FG can improve the LPS-induced inflammatory microenvironment, regulate ECM metabolic disorders of nucleus pulposus cells and aggregation of the NLRP3 inflammasome in vitro, and inhibit cell pyroptosis. Furthermore, in vivo experiments, rhein@FG effectively prevented needle puncture-induced intervertebral disc degeneration in rats.

**Conclusions:**

Rhein@FG has better efficacy than rhein or FG alone due to its slow release and mechanical properties, which can be used as a potential replacement therapy for intervertebral disc degeneration.

**Graphical Abstract:**

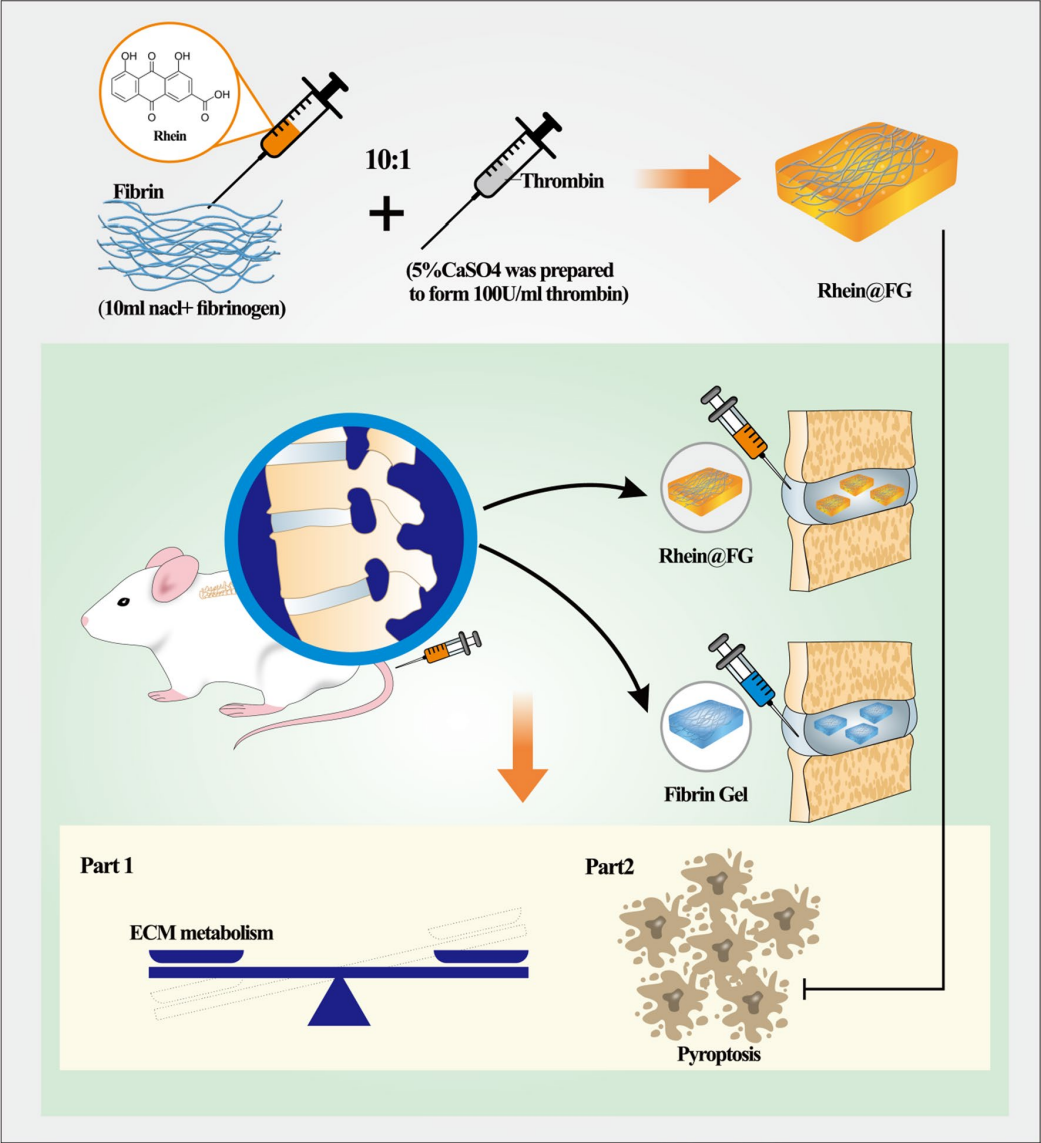

## Introduction

Chronic low back pain (CLBP) is a global social and economic problem, with a prevalence of approximately 1.4–20% [1]. The disability caused by CLBP affects people's daily work and lives [2], with an annual societal cost of 100 billion dollars, which is the main factor causing the economic burden [3, 4]. CLBP is mainly related to intervertebral disc degeneration (IVDD) [5, 6], which is affected by factors such as genetic influences, ageing, mechanical stress, and trauma [7]. Although cell senescence and death are inevitable with age, the emergence of premature senescence is a real problem that has recently attracted attention [8]. Pyroptosis, a newly discovered form of inflammatory programmed cell death, is triggered by the NLRP3 inflammasome [9, 10]. It contributes to the progressive loss of extracellular matrix (ECM), resulting in cell senescence and death [11]. Therefore, the current study suggests that addressing inflammation is an effective way to inhibit cell pyroptosis and delay IVDD [12].

Drug delivery systems are widely used for treating a variety of diseases [13], with advantages such as targeting, high biocompatibility and controlled release [14, 15]. Fibrin glue (FG), this drug delivery vehicle, is composed of fibrinogen solution and thrombin solution rich in calcium [16], and it is used in hemostasis [17], wound healing [18], and bone regeneration [19]. Research has shown that FG, when used as a scaffold, enhances cell survival, proliferation, differentiation, and matrix synthesis, which thereby repairs cartilage lesions and enhances the efficacy of the carrier and is a promising drug delivery system [20].

Rhein is an anthraquinone obtained from the herbal medicine, rhubarb [21]. Rhein is considered to be the main ingredient contributing to anti-inflammatory effects [22]. Although the efficacy of rhein has been confirmed, some factors hinder its clinical application. Rhein has a degree of hepatorenal toxicity [23], studies have shown that 1 mg/ml of rhein can inhibit liver cell proliferation and induce cell apoptosis through oxidative stress, while animal studies have shown that the minimum lethal dose of rhein is 2000 mg/kg [24], indicating that improper use may cause harmful effects to patients. Second, the high hydrophobicity of rhein leads to its low bioavailability [25]. Thus, Rhein-bound fibrin glue loads are expected to overcome therapeutic challenges.

This study examines the characteristics and properties of rhein–fibrin glue (rhein@FG). Furthermore, in vitro inflammatory model used lipopolysaccharide (LPS)-stimulated nucleus pulposus cells (NPCs). Finally, needle puncture-induced IVDD in rats was tested to observe the curative effect of rhein@FG in vivo. We first reported the application of rhein@FG in intervertebral disc degeneration.

## Materials and methods

### Experimental materials

Bovine fibrinogen (340 kDa, 50–70% protein content) was purchased from Yeasen Biotechnology Co. Rhein (purity > 99%) was purchased from MedchemExpress (MCE, USA).Lyophilizing thrombin powder was obtained from Hacon Pharmaceutical Co., Ltd. (Zhejiang, China). Deionized water was used throughout this study.

### Synthesis of rhein@FG

Deionized water was used to prepare fibrin solution with a concentration of 3.5 mg/ml. Rhein was dissolved with dimethyl sulfoxide and mixed with fibrin solution in a syringe. Based on previous reports and preliminary experiments, we ultimately selected a concentration of 2.8 mg/ml of rhein. Then, 100 U of thrombin was added to a 5% calcium sulphate solution, injected into the fibrin solution containing rhein at a ratio of 1:10, and incubated for 2–3 min to obtain rhein@FG.

### Structure and rheological analysis of rhein@FG

A scanning electron microscopy (SEM) image of the external surface of FG and rhein@FG was obtained on HITACHI SU8010. We also observed the surface roughness and morphology using atomic force microscopy (AFM, Bruker Dimension ICON, USA). Rheological properties of the samples were measured on a rotary rheometer (HAAKE MARS60, Germany), and we carried out temperature scanning measurements (temperature: 4–50 °C) and strain scanning measurements (strain: 0.01–100%, frequency: 1 rad/s). Rhein particle sizes and zeta potentials were measured using a nanoparticle size analyser (DLS, Malvern Zetasizer Nano ZS90).

### Drug release

Rhen@FG was added to phosphate-buffered saline (PBS, pH 7.4, 10 mmol/L, 37 °C) in a volume of 5 mL. Two milliliters of sample was regularly absorbed from the sample bottle (1–15 days), and 2 mL of fresh PBS was added to maintain a constant volume. The rhein absorbances were measured at 228 nm using an ultraviolet spectrophotometer (Thermo Scientific, USA). The absorbance value determined by the sample was compared with the absorbance of the mother liquor to calculate the percentage of rhein release.

### Cell culture

NPCs were isolated from the tail discs of 4-week-old male Sprague Dawley rats using nucleus pulposus tissue. We cultured the cells in Dulbecco's modified Eagle's medium (DMEM) with 10% FBS in a humidified incubator with 5% CO_2_.

### Cytotoxicity and proliferation test

NPCs were seeded in 96-well plates (1 * 10^4^/well) and exposed to different concentrations of rhein (5, 15, 25, and 50 µM) for 24 h. Next, 10 µL of CCK−8 solution (Solarbio, China) was added to the cells. Then, the samples were incubated at 37 °C for 2 h. Utilizing a Varioskan Lux microplate reader (Thermo Scientific, USA), absorbances at 450 nm were detected. To further test the cell proliferation abilities after exposure to rhein, FG, and rhein@FG, cell viabilities were examined at 1, 3, and 5 days. Cells treated with rhein, FG, or rhein@FG were subjected to calcein AM staining (Beyotime, China) and counted under a microscope.

### LPS-induced pyroptosis and drug treatment

NPCs were seeded in 6-well plates (3–4 * 10^5^/well) and divided into 5 groups: the CON group, LPS group (LPS treated 23 h + ATP challenge 1 h), rhein group (LPS treated 23 h + ATP challenge 1 h + rhein), FG group (LPS treated 23 h + ATP challenge 1 h + FG), and rhein@FG group (LPS treated 23 h + ATP challenge 1 h + rhein@FG). Cells in all groups were pretreated with the drug for 2 h before adding 2 µg/ml LPS and 5 mM ATP. The concentration of rhein in group of rhein and rhein@FG is both 25 µmol/L. After cell treatments, the samples from each group were processed accordingly for the next test.

### Real-time quantitative (RT-qPCR)

The RNA extracted from lysed NPCs was purified using RNA Purification kit (CW0581, Kangwei) and reverse transcribed with a strand cDNA synthesis kit (Yisheng, China). Then, cDNA was quantified for RT-qPCR. GAPDH served as an internal reference. The primers used are listed in Table 1.

**Table 1:** Primer sequence

Gene	Primer sequence (5′–3′)
Rat Type II collagen A1-F	AAGGGACACCGAGGTTTCACTGG
Rat Type II collagen A1-R	GGGCCTGTTTCTCCTGAGCGT
Rat Aggrecan-F	CAGTGCGATGCAGGCTGGCT
Rat Aggrecan-R	CCTCCGGCACTCGTTGGCTG
Rat Sox9-F	CGGAACAGACTCACATCTCTCC
Rat Sox9-R	GCTTGCACGTCGGTTTTGG
Rat ADAMTS5-F	CCCAGGATAAAACCAGGCAG
Rat ADAMTS5-R	CGGCCAAGGGTTGTAAATGG
Rat NLRP3-F	CAGACCTCCAAGACCACGACTG
Rat NLRP3-R	CATCCGCAGCCAATGAACAGAG
Rat Caspase-1-F	TGCCTGGTCTTGTGACTTGGAG
Rat Caspase-1-R	ATGTCCTGGGAAGAGGTAGAAACG
Rat IL-1β-F	CACCTCTCAAGCAGAGCACAG
Rat IL-1β-R	GGGTTCCATGGTGAAGTCAAC
Rat GSDMD-F	CCAGCATGGAAGCCCTTAGAG
Rat GSDMD-R	CAGAGTCGAGCACCAGACAC
Rat GAPDH-F	CTCATGACCACAGTCCATGC
Rat GAPDH-R	TTCAGCTCTGGGATGACCTT

### Western blot analysis

NPCs were washed three times with PBS and lysed with ice-cold RIPA lysis buffer containing PMSF (1:100) (Fudebio, China).After resting on ice for 15 min, each well was scraped with a cell scraper, and the RIPA was collected into EP tubes, the supernatants of the whole-cell extracts were collected after incubation at 12,000 rpm for 15 min at 4 °C for 15 min, and the protein concentrations were determined using an Enhanced BCA Protein Assay kit (Beyotime, China). One step glue (Fudebio, China) was used, and 10-µL samples along with 10 µL of marker (Fudebio, China) were added to each well. A membrane was transferred to and blocked for 2 h with 5% skim milk before incubation with primary antibodies: rabbit anti-Col2 (1:1000, Proteintech), rabbit anti-Aggrecan (1:1000, Abcam), rabbit anti-Sox9 (1:2000, Proteintech), rabbit anti-ADAMTS5 (1:1000, Proteintech), rabbit anti-NLRP3 (1:500, Proteintech), rabbit anti-Caspase-1 (1:1000, Proteintech), rabbit anti-GSDMD (1:2000, Proteintech), rabbit anti-IL-1β (1:2000, Proteintech), and rabbit anti-GAPDH (1:5000, Proteintech) at 4 °C overnight. TBST is then washed three additional times for 5 min to remove excess antibodies, For 1 h at room temperature, specific horseradish peroxidase-conjugated secondary antibodies (Beyotime, China) were incubated with the membrane. Subsequently, excess secondary antibodies were removed by three 5-min TBST washes. ChemiDoc touch imaging system (Bio-Rad, USA) was used to measure the signal intensity of the reaction band on the membrane. All of the above experiments were carried out at three different times and repeated three times.

### Immunofluorescence

Fixed cells were washed three times with PBS and fixed with precooled methanol for 15 min at − 20 °C. The cells were washed three times with PBS again after permeating with 0.5% Triton X-100 for 20 min. We blocked the cells with 5% BSA for another 30 min and added primary antibodies and incubated overnight at 4 °C. We added secondary goat anti-rabbit Alexa Fluor 488 and 594 antibodies (Beyotime, China) after three washings with PBS. For 10 min, samples were stained with DAPI (Beyotime, China) after 1 h at room temperature. The above experiments were performed three times and repeated three times using a fluorescence microscope (Olympus BX51, Japan).

### Needle puncture-induced IVDD in rats

In this study, 8-week-old male Sprague–Dawley (SD) rats weighing approximately 200 g were selected for the in vivo experiments. All rats were purchased from the animal ethics committee of Zhejiang Chinese Medical University. Rats were randomly divided into four groups: puncture, rhein, FG and rhein@FG group (*n* = 3 per group). The animals were anesthetized with ketamine and xylazine (10:7100 mg/kg) intraperitoneally. IVDD was established by puncturing the coccygeal space at C6-7, C7-8, and C8-9. After disinfection with iodoalcohol, a 26G needle (diameter = 0.45 mm) was inserted at the level of the annulus fibrosus by palpation and passed through the NP to reach the contralateral annulus fibrosus. After incomplete penetration, the needle was rotated 360° twice for 30 s. All experiments were conducted under sterile conditions. One week after the initial puncture, each group of rats was injected with a 26G needle for treatment: puncture group (intradiscal injection of 5 µL PBS), rhein group (intradiscal injection of 5 µL rhein), FG group (intradiscal injection of 5 µL FG) and rhein@FG group (intradiscal injection of 5 µL rhein@FG). C5-6 segments of all rats served as controls and served as the NC group. All SD rats were reared for 4 and 8 weeks.

### Radiological assessment analysis

The rats in each group were examined by micro-CT (SkyScan, Belgium) and MRI (Universal Corporation, USA) at 4 and 8 weeks, respectively, to detect the changes in height of the caudal intervertebral space and the signal intensities of the nucleus pulposus of the intervertebral discs. ImageJ (National Institutes of Health, USA) was used to measure the disc heights in the CT images and to quantify the T2-weighted signal intensities of the disc nucleus pulposus.

### Histological evaluation of IVDD

The rats in each group were killed by excessive carbon dioxide at the 4th and 8th weeks. We removed the corresponding intervertebral disc segments and temporarily fixed them in 10% neutral buffered formalin. Skin and muscle tissues were removed by dissection, and the samples were then immersed in a decalcifying solution (14% EDTA) for decalcification for 4 weeks. H&E(HE), safranin O-fast green(SO/FG), and Masson staining of 8 μm tissue sections were performed, and photographs were obtained with a digital scanning microscope to analyse the pathological and structural changes of the intervertebral disc nucleus pulposus and fibrosis. Histological images of each group were graded from 0 to 15 points according to histological grade (Table 2) by an investigator blinded to the specific grouping.

**Table 2:** Histological grade

Category	Grade
I. Cellularity of the annulus fibrosus	1. Fibroblasts comprise more than 75%
	2. The proportion of both fibroblasts and chondrocytes does not exceed 75% cells
	3. Chondrocytes comprise more than 75%
II. Morphology of the annulus fibrosus	1. Well organized collagen layer without rupture or serpentine fibres
	2. Bulging inward, ruptured or serpentine fibres less than one-third
	3. Bulging inward, ruptured or serpentine fibres more than one-third
III. Border between the annulus fibrosus and nucleus pulposus	1. Normal
	2. Minimal interruption
	3. Moderate or severe interruption
IV. Cellularity of the nucleus pulposus	1. Normal
	2. The quantity has slightly decreased. There are some aggregated cells
	3. Moderate or severe reduction (50%). Cells either aggregate or disperse
V. Morphology of the nucleus pulposus	1. Round, accounting for at least half of the intervertebral disc area
	2. Rounded or irregularly shaped, including quarter to half of the intervertebral disc area
	3. Irregular in shape, less than one-fourth of the area of the intervertebral disc area

### Statistical analysis

In this study, all data are presented as the mean ± standard deviation. Data were analysed using a one-way ANOVA. SPSS 23.0 was used to analyse the data. *P* < 0.05 indicates statistical significance.

## Results

### Morphology and characteristics of rhein@FG

**Fig. 1:**
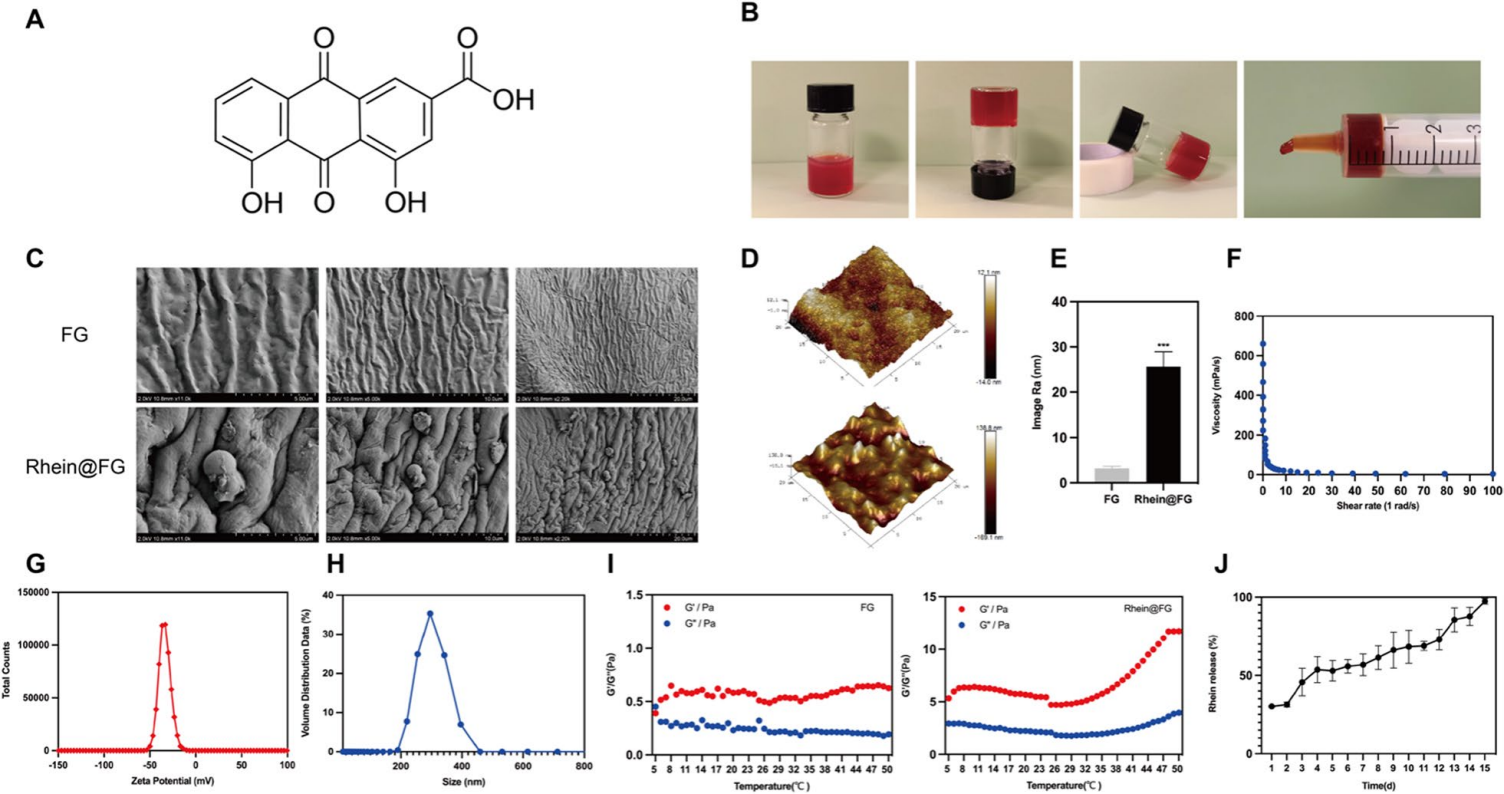
Morphology and characteristics of rhein@FG. **A** Molecular formula of Rhein. **B** Hydrogel status and injected images. **C** SEM showed the morphologies of FG and rhein@FG. **D**, **E** 3D height morphologies and relative height roughness (Ra) values of rhein@FG (*n* = 3 *P < 0.05). **F** Shear rates of rhein@FG. **G**, **H** The zeta potentials and particle sizes of rhein@FG. **I** Rheology of FG and rhein@FG. *G*′ is the storage modulus, and *G*″ is the loss modulus. **J** Rhein@FG exhibited sustained release properties

As shown in Figure, rhein@FG has a uniform saffron appearance and can be expelled by a syringe and maintained in gel form (Fig. 1B). The rheological property evaluation indicates that the viscosity of rhein@FG decreases with increasing shear rate, which further indicates that it has good injectability (Fig. 1F). SEM observations detected the surface morphology of rhein@FG when it was dry. We clearly observed that rhein was wrapped and attached to fibrin glue (Fig. 1C). We used AFM to further observe the surface morphology and roughness of rhein@FG. As shown in the 3D height morphology (Fig. 1D), the relative height roughness (Ra) of rhein@FG is higher than that of FG, which means that inclusion of rhein on the surface increases the roughness of the gel surface (Fig. 1E). DLS showed that the average particle size of rhein was 484.5 nm, with a negative zeta potential of − 33.8 mV, which means that rhein can firmly bind to FG (Fig. 1G, H). Furthermore, compared with the FG group, we found that rhein@FG has better viscosity and stability in temperature scanning, rhein@FG shows that the storage modulus (*G*′) is greater than the loss modulus(*G*″), which indicates that the gel can maintain a colloidal state at 37 °C in vivo, which can provide certain mechanical properties (Fig. 1I). Finally, we simulated rhein@FG release in vivo. The results showed that the release process was slow in the whole stage, and approximately 90% could be released by the 14th day, indicating the slow-release property of rhein@FG.

### Rhein@FG had no effect on NPC survival and proliferation in vitro

**Fig. 2:**
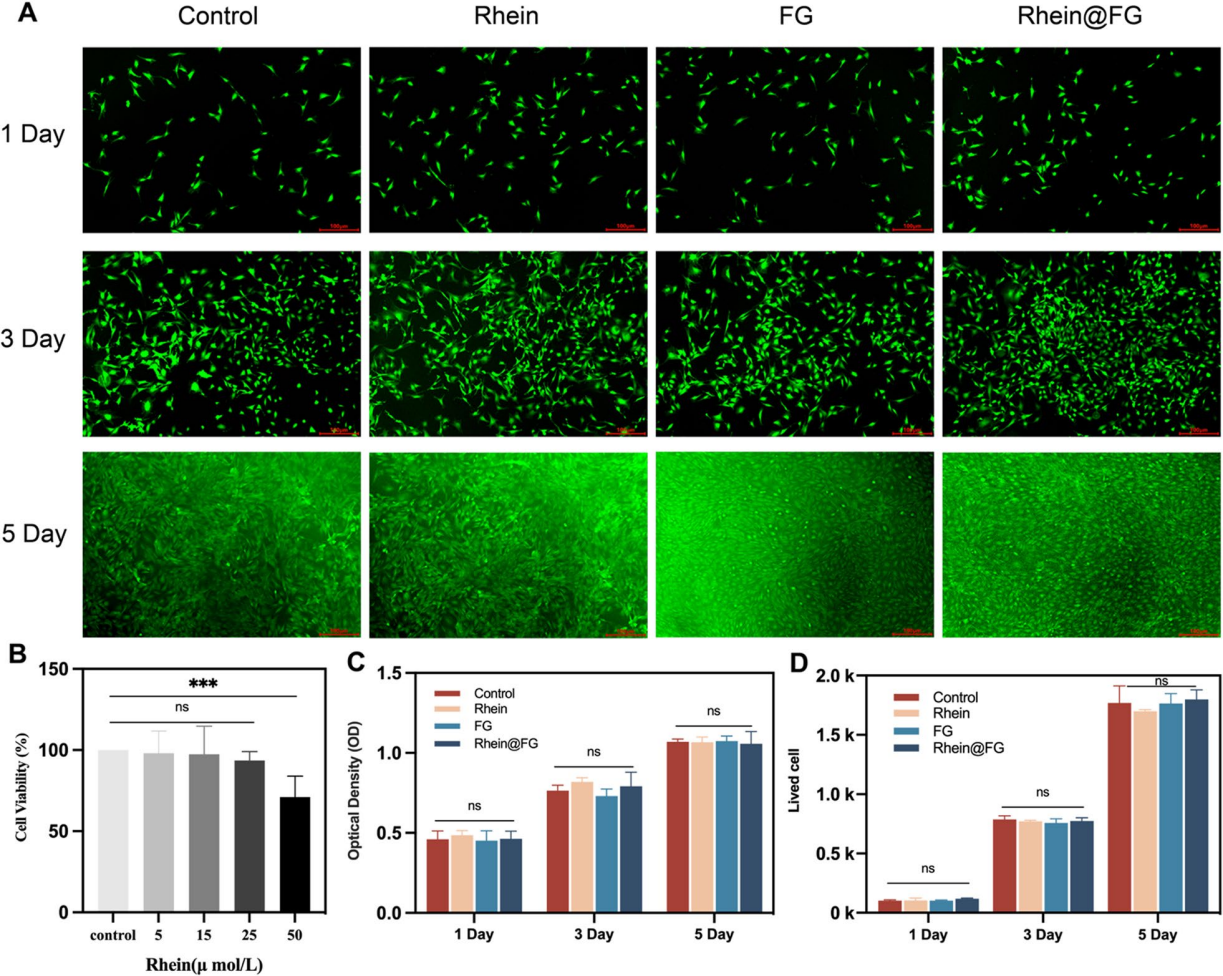
Biocompatibility of rhein@FG. **A** Image of living cells (green) in each group. Scale bars = 100 mm. **B** Viabilities of cells cultured with rhein. **C** Cell proliferation test. **D** Cell counts of living cells (*n* = 3 *P < 0.05)

Rhein cytotoxicity was evaluated by determining the cell viabilities of NPCs. As for CCK8, the results showed that when measured in the range of 0–25 μM, there was no apparent effect, whereas at 50 μM, the viability decreased significantly, which was significantly different from the other concentrations (Fig. 2B). According to the results for CCK8, 25 μM was selected as the intervention concentration for subsequent cell experiments. CCK8 and live cell staining were used to observe cell proliferation in rhein, FG and rhein@FG (Fig. 2A). There were no significant effects on cell proliferation on Days 1, 3 and 5, indicating that rhein@FG has good biocompatibility and that FG is an ideal carrier for rhein (Fig. 2C, D).

### Rhein@FG regulates ECM metabolism in NPCs

**Fig. 3:**
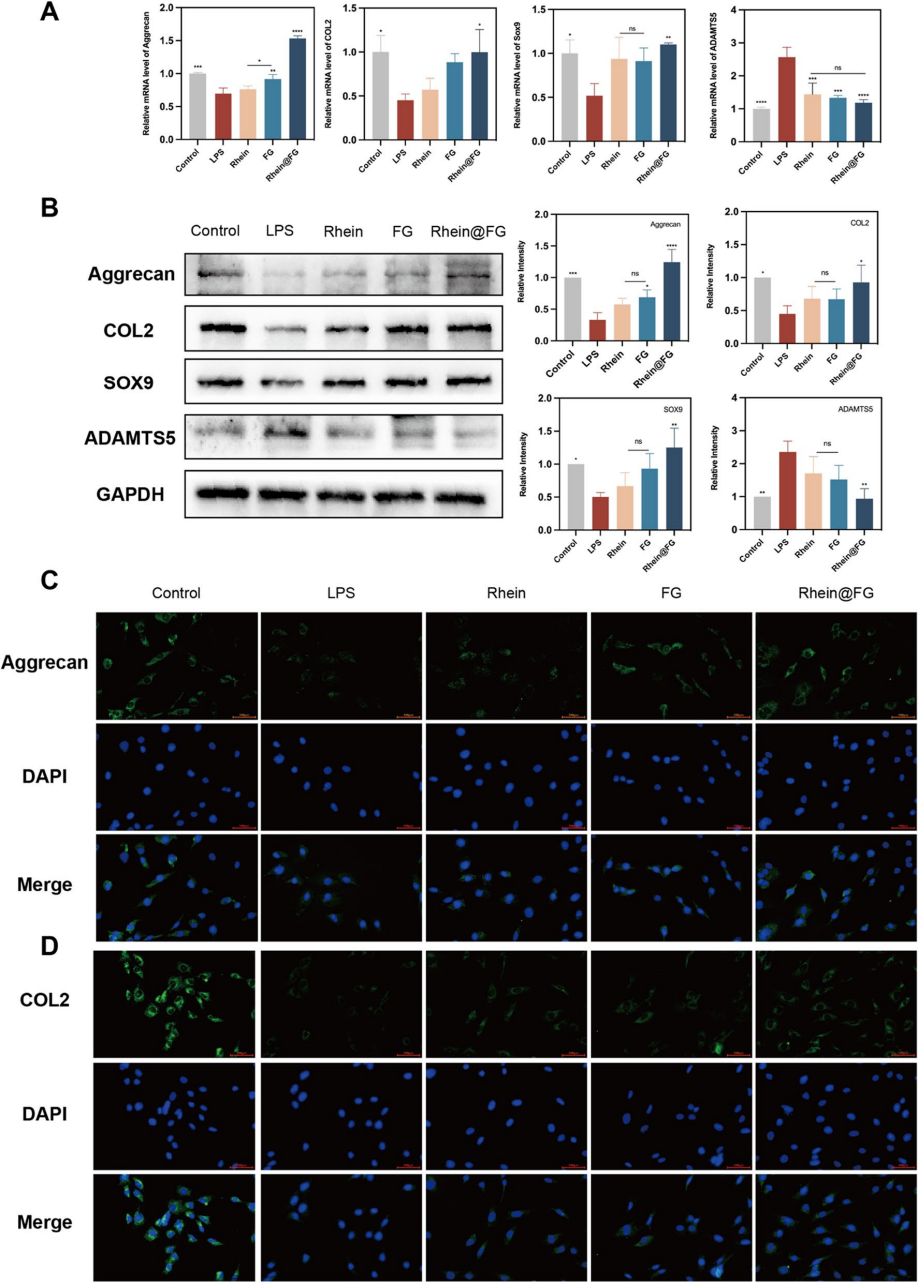
mRNA expressions of NPCs by real-time quantitative PCR. **A** Rhin@FG regulates the matrix metabolism of NPCs. **B** Protein expression levels of Aggrecan, Col2, Sox9, and ADAMTS5 while GAPDH serves as a loading control. *P < 0.05. **C**, **D** Fluorescence images of Aggrecan and Col2. Scale bars = 100 μm

To evaluate the effect of rhein@FG gel on ECM regulation, we first used RT‒qPCR to detect the expressions of genes related to the synthesis and degradation in LPS-induced rat NPCs. LPS stimulation significantly decreased the expression of aggrecan, Col2 and Sox9 in nucleus pulposus cells compared to the control group (Fig. 3A). Fortunately, drug intervention can improve this situation, and it is worth noting that the rhein@FG group showed better efficacy. Moreover, drug treatment inhibited the LPS-induced increase in ADAMTS5 expression to different degrees. However, there were no significant differences between the groups of drugs (*P* > 0.05). To confirm the RT-qPCR results, we also used Western blotting (Fig. 3B) and found that stimulation with LPS increased the expressions of catabolic proteins and inhibited synthesis, as shown in the LPS group. At the same time, we found that rhein@FG gel treatment can reverse this trend and has better efficacy than rhein or FG alone. The expressions of Aggrecan and Col2 improved in the rhein@FG group compared to the LPS group (*P* < 0.05), as shown by the fluorescence intensities, which again demonstrated the therapeutic advantage of gel-containing drugs. These data were generally similar to the RT-qPCR results. Furthermore, based on the Aggrecan and Col2 immunofluorescence results (Fig. 3C), we once again proved the influence of LPS on ECM metabolism in NPCs and the therapeutic effect of drugs. Therefore, these results suggest that rhein@FG gel can affect the expressions of metabolic genes involved in ECM synthesis and catabolism and shows advantages over a single drug in regulating changes in the microenvironment during the early stages of IVDD.

### LPS-induced pyroptosis in NPCs is inhibited by rhein@FG

**Fig. 4:**
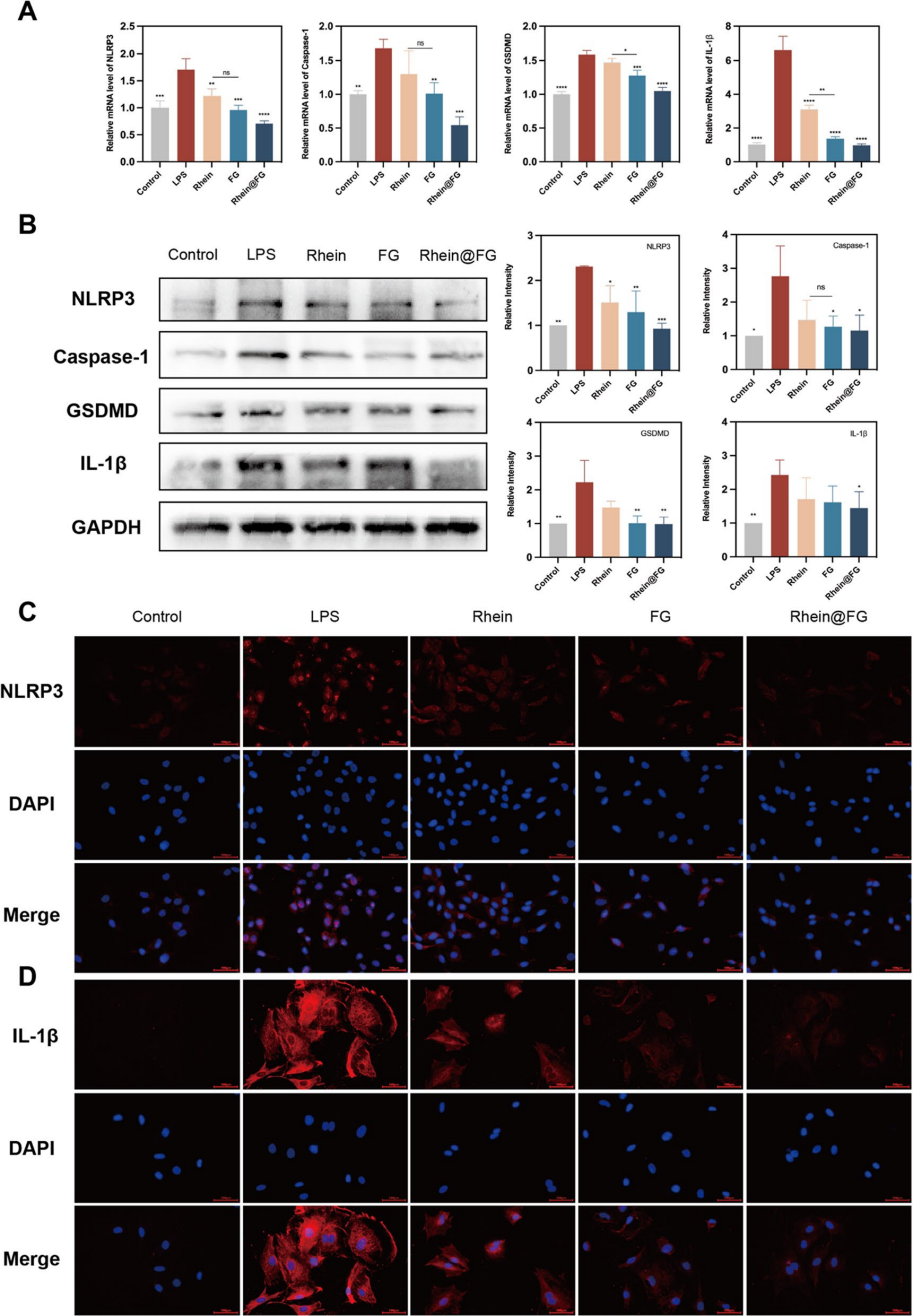
The Rhein@FG modulated pyroptosis in NPCs. **A** mRNA expression in NPCs. **B** A quantification of NLRP3 protein expression levels was done by measuring GAPDH levels as a loading control. Data are presented as means and standard deviations (*n* = 3). **C**, **D** A fluorescence image of NLRP3 and IL-1β. Scale bar = 100 μm

In addition, to demonstrate that rhein@FG gel inhibits inflammation to alleviate the IDD pathway, we hypothesized that it could reduce pyroptosis by inhibiting the expression of the NLRP3 inflammasome. After LPS stimulation, NLRP3, Caspase-1, GSDMD and IL-1β expression were significantly upregulated (*P* < 0.05). A better inhibitory effect was found in NPCs exposed to the rhein@FG group than in the positive control group (Fig. 4A). Thus, rhein@FG might protect NPCs by inhibiting the pyroptosis pathway. Moreover, we evaluated NPCs pyroptosis at the protein level. Different groups were exposed to the drug and their protein levels were measured in the LPS group and in groups exposed to the drug and the control group. The protein levels increased in the LPS group, but the groups exposed to rhein and/or FG exhibited reduced protein levels. Significant differences (*P* < 0.05) were noted based on quantitative analysis (Fig. 4B). In addition, as depicted in Fig. 4C, D, the immunofluorescence results showed that the group treated with rhein@FG had significantly lower fluorescence intensity than that of the group treated with LPS. These results indicate that rhein and FG inhibit the expressions of key proteins in the pyroptosis pathway to decrease the extent of pyroptosis in NPCs and that FG loaded with rhein has a better inhibitory effect.

### Rhein@FG delayed needle puncture-induced IVDD in rats in vivo

**Fig. 5:**
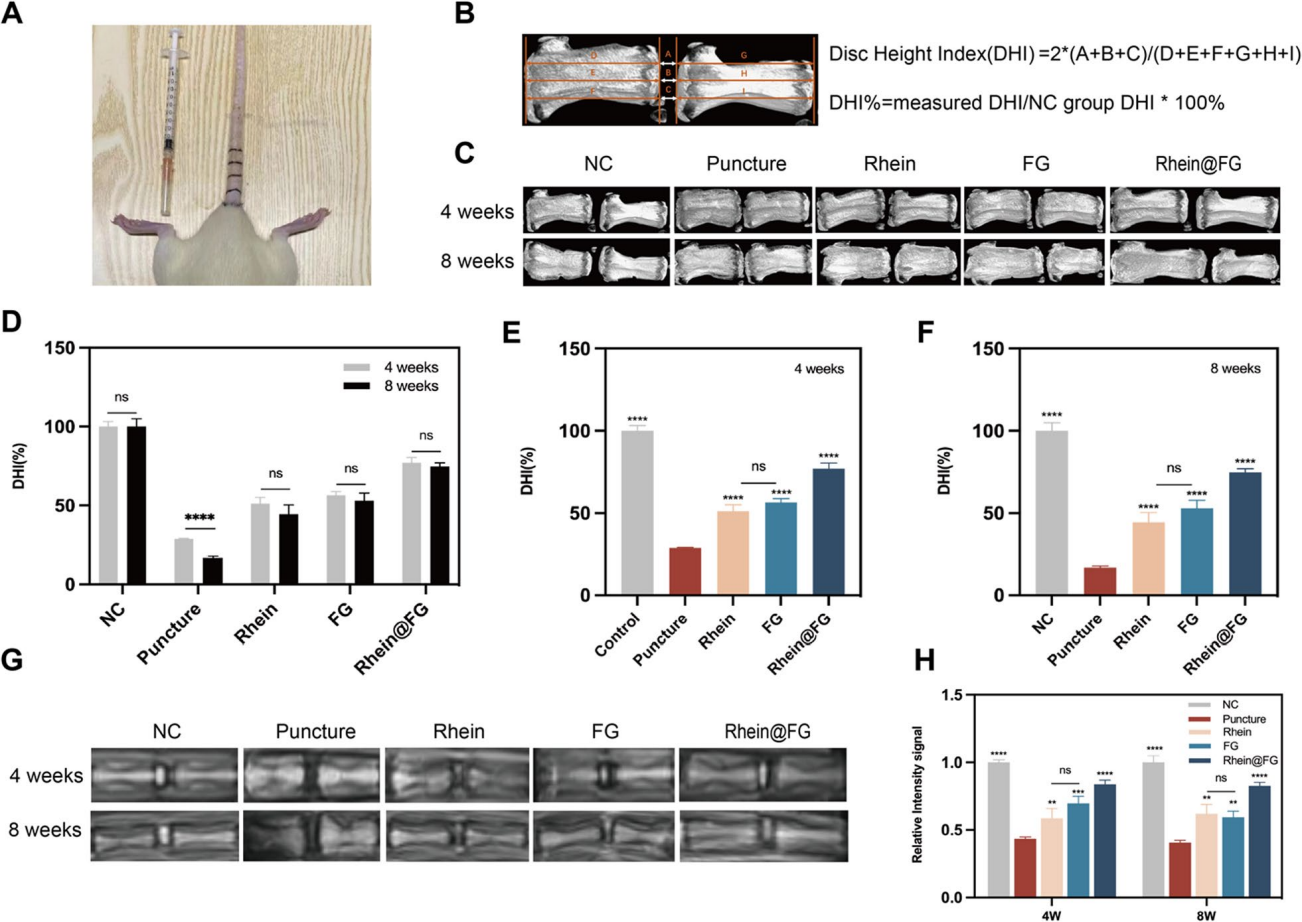
In addition to improving the nucleus pulposus signal intensity, Rhein@FG can maintain the mechanical properties of intervertebral discs. **A** Puncture modelling IVDD view. **B** DHI% calculation method. **C** CT was used to observe the differential changes in intervertebral disc space height in rats. **D**–**F** Statistically different changes in disc space heights. **G** T2-weighted signal intensities of the nucleus pulposus of rat intervertebral discs were measured with MRI. **H** The nucleus pulposus T2-weighted signal intensities were counted. Data are presented as the SD ± mean (*n* = 8) *P < 0.05

In order to explore the repair effect of rhein@FG gel on IVDD in rats, we established a model based on needle punctures in the coccyx discs (Fig. 5A). Then, the drug is injected into the intervertebral disc. The disc height index was measured in CT images using Data Viewer software (Fig. 5B). At 4 weeks after surgery, the height of the intervertebral spaces in the rats began to decrease (Fig. 5C). A significant difference was found between the NC group and the modelling group (*P* < 0.05). After injections of rhein and/or FG for 4 weeks, this decreased height of the intervertebral spaces was still highly preserved in the treatment group compared to the modelling group (*P* < 0.05) (Fig. 5E). Despite a reduction in height in each group after 8 weeks of injection treatment, the heights of the treatment group were significantly different from the model group (*P* < 0.05) (Fig. 5D, F). In addition, at 4 and 8 weeks after injection (Fig. 5G), rats in the NC group showed higher T2-weighted signals, indicating sufficient water content. There was significant degeneration of the nucleus pulposus in the puncture model group compared to the control group (*P* < 0.05), and the intensities of the nucleus pulposus signals in the drug intervention group improved to different degrees compared to the puncture model group (*P* < 0.05), among which rhein@FG group was the most effective (Fig. 5F). Thus, according to the results, the degree of nucleus pulposus degeneration was decreased after injections with rhein@FG treatment, thereby facilitating maintenance of the height of the intervertebral space.

**Fig. 6:**
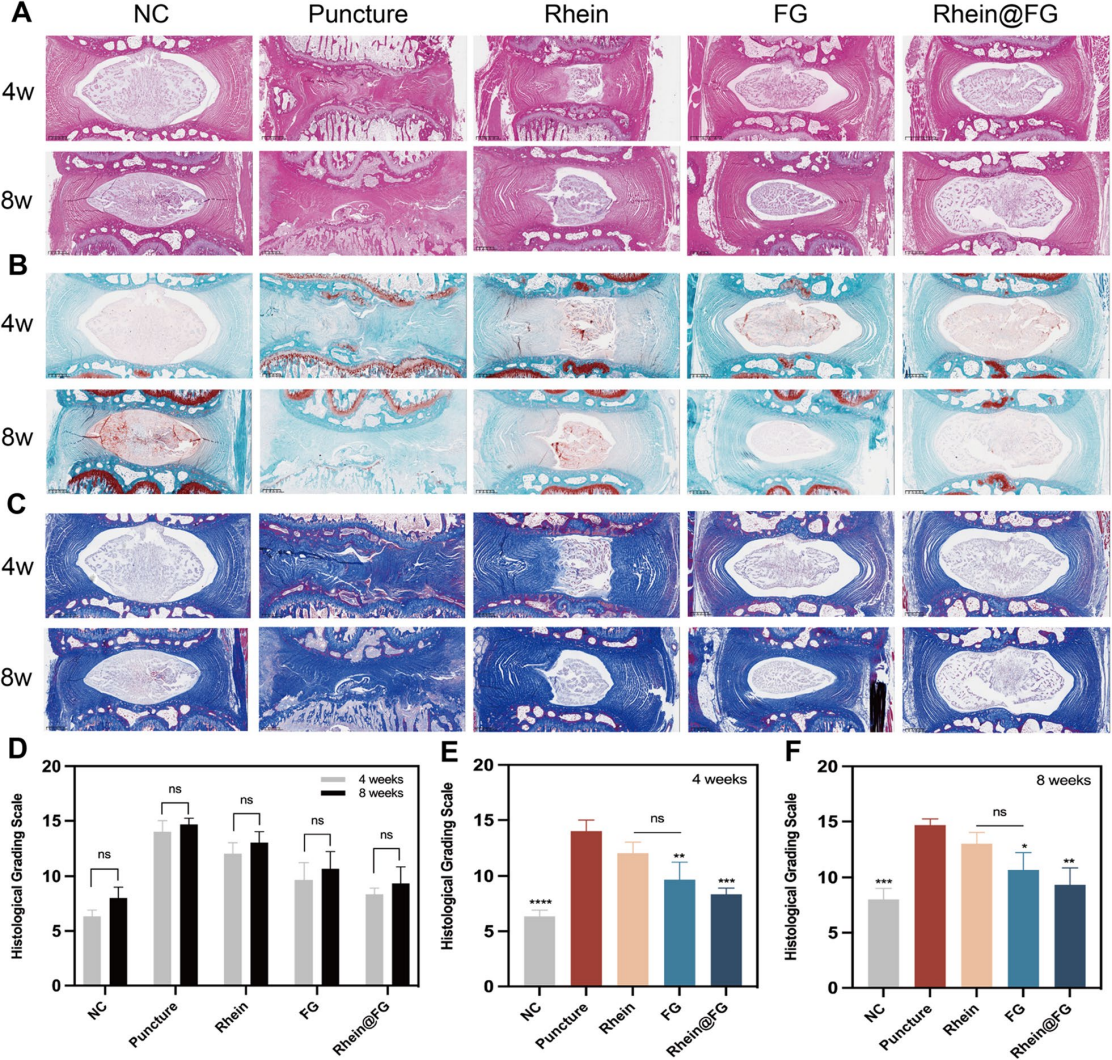
Histological evaluations of animal experiments. **A** 4 and 8 weeks HE staining images of rat intervertebral discs. **B** OS/FG stain. **C** The degree of IVDD progression was assessed based on the morphology and cellularity of the NP and AF in Masson staining. At 4 and 8 weeks, histological grade changes in each group are presented as SD ± mean (*n* = 8) *P < 0.05

We further evaluated disc degeneration using different staining methods at 4 and 8 weeks to study the effect of FG and/or rhein on IVDD repair in vivo. Assessments using HE, Masson and SO/FG staining (Fig. 6A–C) revealed a relatively complete nucleus pulposus and fibrous ring structure, a relatively even distribution of cells, no fibre breaks, and no serpentine appearance in the control group from 4 to 8 weeks. There was a large degree of rupture and shrinkage in the nucleus pulposus in both the puncture model and treatment groups. The puncture model group even had large areas exhibiting collapse and degeneration of the cartilage endplates. However, in the treatment group, disc tissues were repaired to a greater extent than in the puncture model group. The nucleus pulposus volumes were more preserved in the treatment group than in the puncture model group. Most of the cells showed homogeneous distributions, and the annulus fibrosus structures and heights were also largely preserved. We scored the samples obtained at 4 and 8 weeks based on IVDD histological evaluations. The puncture model group received the highest score, and the scores for the treatment groups were significantly different from that of the puncture model group (*P* < 0.05) (Fig. 6D–F). OS staining also showed that the treatment groups had darker colours of safranin staining than the puncture model group. These results demonstrated that rhein@FG gel might delay disc degeneration by inhibiting local inflammation, maintaining the balance of ECM synthesis/degradation in disc NPCs, and promoting tissue repair.

## Discussion

It is important to maintain spinal health and activity by maintaining the intervertebral disc, which includes the nucleus pulposus, the annulus fibrosus, and the cartilage endplates [26]. Nucleus pulposus cells are chondrocytes that are able to synthesize and secrete functional ECM such as type II collagen and aggrecan, thus playing a key role in maintaining structural stability and functional integrity of the intervertebral disc [27]. A healthy ECM maintains an equilibrium between synthesis and catabolism. However, when the intervertebral disc is subjected to an inflammatory microenvironment, this balance is broken, which is mainly manifested by the upregulation of metalloproteinases, ADAMTS4, ADAMTS5, MMP3, and MMP13 [28]. In addition, ECM degradation occurs as Col2 content decreases due to decreases in synthetic proteins, such as Sox9 [10, 29]. In this study, we produced a fibrin gel containing rhein, which can improve the inflammatory microenvironment, regulate the ECM metabolic balance of intervertebral discs and treat intervertebral disc degeneration.

Rhein exhibits excellent anti-inflammatory properties [30] and can stimulate NF-κB signaling via a variety of mechanisms [31], MAPK signaling pathway [32], and PI3K/AKT pathway [33]. Ge et al. [34] demonstrated that rhein inhibits the production of proinflammatory factors induced by LPS and significantly reduces the phosphorylation of NF-κb p65 and inducible NO synthase, as well as the expression of Cox-2. In addition, inflammasome pathways NF-κB and NLRP3 are inhibited by rhein, contributing to its anti-inflammatory action. According to Li et al. [35], rhein inhibited IL-1β-induced secretion of MMPs and aggrecanases and is a potential biological therapy for IVDD. Similarly, we found in this study that rhein can inhibit LPS-induced NLRP3 aggregation and thus inhibit pyroptosis and restore the balance of ECM metabolism in NPCs.

It is generally believed that the effectiveness of medical treatments can be greatly improved by means of controlled delivery of biomaterial carriers. Biomaterials are recognized for their biocompatibility, biological functionality and bioabsorbability in vivo [36]. Yin et al. [37] incorporated rhein into a silk fibroin hydrogel to treat infected wounds, which has sustained anti-inflammatory and antibacterial effects. Zhou et al. [38] produced a rhein-chitosan hydrogel, which showed a time-dependent anti-neuroinflammatory effect. We solved the problem of the low bioavailability of rhein by carrying rhein with fibrin glue. A good biodegradability makes FG a valuable therapeutic strategy in tissue engineering [39]. In our study, both FG and rhein@FG was injectable and showed good biocompatibility in vitro. Previous studies have found that the presence of FG has a positive impact on Col2 and Aggrecan expressions, which contributes to the survival and proliferation of chondrocytes [40–43]. Furthermore, FG enlists anti-inflammatory cytokines and enhances anti-inflammatory effects [44, 45]. Similar to current research, we have noticed that the FG group exhibits certain therapeutic effects in maintaining ECM balance and anti-inflammatory effects. Surprisingly, the inclusion of rhein in FG enhanced the therapeutic effect, promoted ECM metabolism of NPCs to a greater extent, enhanced the anti-inflammatory effect of rhein, inhibited NLRP3 aggregation, and thus prevented the occurrence of cell pyroptosis. In addition, rhein@FG also showed a better therapeutic effect in vivo. On the one hand, its therapeutic advantage may be due to its sustained release characteristics, and the mechanical properties provided by FG are conducive to delaying IVDD [46]. The final result shows that FG containing rhein has an excellent anti-inflammatory effect. Continuous rhein release improves the local microenvironment, inhibits cell pyroptosis, and then delays IVDD.

Nevertheless, this study still has some limitations. Due to objective conditions such as experimental facilities, this study did not observe whether rhein@FG alleviated the pain caused by intervertebral disc degeneration in rats through its anti-inflammatory effect. In addition, this study fell short of identifying the precise mechanism by which rhein@FG suppresses NLRP3 aggregation and pyroptosis. The specific molecular mechanism of rhein@FG delaying IVDD deserves further exploration.

## Conclusions

In this study, a new type of rhein@FG gel was developed with good mechanical strength, sustained release, and low toxicity. Rhein loading into FG provides a continuous anti-inflammatory effect, inhibits pyroptosis of NPCs by inhibiting the aggregation of NLRP3, regulates ECM metabolism of NPCs, and repairs IVDD.
